# Comparison of qSOFA score, SOFA score, and SIRS criteria for the prediction of infection and mortality among surgical intermediate and intensive care patients

**DOI:** 10.1186/s13017-020-00343-y

**Published:** 2020-11-25

**Authors:** Christian Koch, Fabian Edinger, Tobias Fischer, Florian Brenck, Andreas Hecker, Christian Katzer, Melanie Markmann, Michael Sander, Emmanuel Schneck

**Affiliations:** 1 Department of Anesthesiology, Operative Intensive Care Medicine and Pain Therapy, University Hospital of Giessen, Rudolf-Buchheim-Street 7, 35392 Giessen, Germany; 2 German Center of Infection Research (DZIF), Partner Site Giessen/Marburg/Langen, Giessen, Germany; 3 Department of General and Thoracic Surgery, University Hospital of Giessen, Giessen, Germany

**Keywords:** Sepsis, Critical care, qSOFA, SOFA, Mortality, Infections

## Abstract

**Background:**

It is crucial to rapidly identify sepsis so that adequate treatment may be initiated. Accordingly, the Sequential Organ Failure Assessment (SOFA) and the quick SOFA (qSOFA) scores are used to evaluate intensive care unit (ICU) and non-ICU patients, respectively. As demand for ICU beds rises, the intermediate care unit (IMCU) carries greater importance as a bridge between the ICU and the regular ward. This study aimed to examine the ability of SOFA and qSOFA scores to predict suspected infection and mortality in IMCU patients.

**Methods:**

Retrospective data analysis included 13,780 surgical patients treated at the IMCU, ICU, or both between January 01, 2012, and September 30, 2018. Patients were screened for suspected infection (i.e., the commencement of broad-spectrum antibiotics) and then evaluated for the SOFA score, qSOFA score, and the 1992 defined systemic inflammatory response syndrome (SIRS) criteria.

**Results:**

Suspected infection was detected in 1306 (18.3%) of IMCU, 1365 (35.5%) of ICU, and 1734 (62.0%) of IMCU/ICU encounters. Overall, 458 (3.3%) patients died (IMCU 45 [0.6%]; ICU 250 [6.5%]; IMCU/ICU 163 [5.8%]). All investigated scores failed to predict suspected infection independently of the analyzed subgroup. Regarding mortality prediction, the qSOFA score performed sufficiently within the IMCU cohort (AUCROC SIRS 0.72 [0.71–0.72]; SOFA 0.52 [0.51–0.53]; qSOFA 0.82 [0.79–0.84]), while the SOFA score was predictive in patients of the IMCU/ICU cohort (AUCROC SIRS 0.54 [0.53–0.54]; SOFA 0.73 [0.70–0.77]; qSOFA 0.59 [0.58–0.59]).

**Conclusions:**

None of the assessed scores was sufficiently able to predict suspected infection in surgical ICU or IMCU patients. While the qSOFA score is appropriate for mortality prediction in IMCU patients, SOFA score prediction quality is increased in critically ill patients.

## Background

Sepsis is defined as a life-threatening disease complex characterized by severe organ dysfunction resulting from a dysbalanced host response to an infection [1]. Despite modern treatment protocols, sepsis-related mortality remains highly associated with delays in adequate treatment [2]. For this reason, modern clinical concepts have focused on the development of criteria aiming for the rapid identification of sepsis [3, 4].

For 24 years, sepsis has been defined as suspected or proven infection, together with two or more systemic inflammatory response syndrome (SIRS) criteria [5]. However, during the last decade, clinical characteristics that serve to define sepsis changed due to an improved understanding of the underlying pathobiology. Therefore, in 2016, the Third International Consensus Definitions for Sepsis and Septic Shock (Sepsis-3) introduced a significant change in the approach to the definition and diagnostic criteria of sepsis [1].

Nevertheless, a highly sensitive and specific diagnostic test for the detection of sepsis is currently still lacking. Among ICU encounters with suspected infection, the Sepsis-3 Task Force recommended the use of the Sequential (sepsis-related) Organ Failure Assessment (SOFA) score for the identification of septic patients [1, 6]. For the rapid identification of patients with suspected infection outside of the ICU, on the other hand, Seymour et al. introduced the quick Sequential Organ Failure Assessment (qSOFA) score [7]. The qSOFA score is a simple score consisting of three items: respiratory rate (RR) ≥ 22 breaths per minute, altered mentation (Glasgow Coma Scale [GCS] < 15), and systolic blood pressure (SBP) < 100 mmHg. A qSOFA score ≥ 2 was found to be significantly predictive of increased all-cause mortality in patients outside of the ICU [7]. Therefore, the authors of the Third International Consensus Definitions for Sepsis and Septic Shock (Sepsis-3) recommended the use of the qSOFA score for the identification of adult septic patients in out-of-hospital, emergency department, or general hospital ward settings [1].

Intermediate care units (IMCUs) are logistically situated between ICUs and general wards and serve as an alternative care setting for patients deemed too unstable to be cared for on the general ward, but without requiring the resources of an ICU [8–10]. Lacking a unitary definition of IMCUs, their nomenclature varies from high dependency, progressive care, medium care, or step-down units, resulting in a high variability of organizational practice [8]. While most IMCUs offer continuous monitoring of vital signs, the ability to provide mechanical ventilation, renal replacement therapy, and differentiated catecholamine therapy is normally limited [11]. Although IMCU patients commonly demand a higher level of nursing compared to the normal ward, the severity of illness is lower than on the ICU [12, 13]. It is worth noting that the mere presence of an IMCU is associated with a significantly reduced hospital mortality in ICU patients, underlining the impact of an IMCU as a bridge between the ICU and the regular ward [14]. Furthermore, by demonstrating that septic shock patients can be successfully treated on an IMCU, Meaudre et al. proposed the potential of this critical care resource [15]. Surgical patients, in particular, are often treated in IMCUs because they are commonly extubated shortly after surgery and are therefore not mandatorily eligible for ICU treatment. However, surgical patients are also at risk for postoperative infections. Clinical signs of infection in these patients are challenging, since they might also be caused by the surgery itself, implicating the need for thorough risk stratification [16, 17]. Lacking evidence, it is not yet defined whether these patients should be evaluated as ICU or non-ICU patients when it comes to the identification of sepsis, respectively severe infection, raising the question as to whether the SOFA or qSOFA score should be used. For this reason, there are currently no specific recommendations for the screening of septic patients treated on IMCU. Therefore, the aim of our study was to compare the predictive power of qSOFA and SOFA scores, as well as the 1992 defined SIRS criteria, for mortality or infection in a large sample of surgical ICU and IMCU patients. We hypothesized that the qSOFA score would perform superiorly to the SOFA score and SIRS criteria in predicting mortality or infection among IMCU patients.

## Methods

### Study design and patient recruitment

This retrospective, 6-year cohort study was approved by the local ethics committee (Justus-Liebig-University, Giessen, Germany, trial code 240/16). The methods and results are presented in accordance with the Strengthening the Reporting of Observational Studies in Epidemiology (STROBE) guidelines. Data of all patients aged ≥ 18 years with suspected infection who were treated at the surgical ICU and/or IMCU of the University Hospital of Giessen between January 01, 2012, and September 30, 2018, were included.

### Data acquisition

After identification of patients, study data were automatically extracted from the local patient data management system (ICU-Data®, IMESO® GmbH, Giessen, Germany) with Structured Query Language and Procedural Language (SQL/PL-SQL)-based scripts. Patients’ characteristics included age, body mass index (BMI), treatment unit (ICU, IMCU, or both), Acute Physiology and Chronic Health Evaluation (APACHE) II score, and, if applicable, the type of performed surgery. Episodes of suspected infections were defined as the first 72 h after starting treatment with broad-spectrum antibiotic agents, which included carbapenems, glycopeptides, quinolones, piperacillin/sulbactam, ceftazidime, cefepime, linezolid, tigecycline, daptomycin, and fosfomycin. Contrarily, the following antibiotics were excluded because they did not meet the definition of broad-spectrum antibiotic treatment, according to the European and local sepsis guidelines [18]: ampicillin, cefazolin, cefuroxime, colistin, metronidazole, erythromycin, trimethoprim/sulfamethoxazole, and azithromycin.

While the SOFA score was recorded daily throughout the patient’s ICU treatment by the attending physician, SIRS criteria and qSOFA score were not registered systematically and therefore needed to be calculated retrospectively. First, relevant vital signs (respiratory rate, systolic blood pressure, heart rate, temperature), which were automatically recorded every 15 min, were systematically analyzed for outliers. For this purpose, a second data table was built, and the median for each parameter was calculated. For the calculation of the median of the respiratory and heart rates as well as the systolic blood pressure, three values of each time point were included (i.e., corresponding time point and two values aside). Since extreme values of both parameters were possible in critically ill patients, no absolute thresholds were defined as outliers. Secondly, the median for each temperature time point was calculated out of seventeen values (i.e., corresponding time point and sixteen values aside) to equalize incorrect measurements, which can be caused by a dislocated temperature probe. Body temperature measurements ≤ 31 °C were defined as artefacts and therefore excluded. If GCS was not available, Richmond Agitation Sedation Scale (RASS) was used for the assessment of consciousness (where RASS ≠ 0 was defined as the fulfillment of “altered mental status,” respectively as GCS ≤ 15). Leucocyte count was derived from the daily routine blood cell count, while arterial carbon dioxide partial pressure (paCO_2_) was extracted from the blood gas analyses, which was most recent to the analyzed time frame.

In accordance with their definitions, the SIRS criteria and the qSOFA score were rated positive if at least two criteria were fulfilled during a minimum of 30 min [7, 19]. The SOFA score of each day was compared with the value of the previous day. An increase of at least two points was rated positive.

Furthermore, the outcome analysis included the need for and duration of invasive ventilation, requirement for catecholamines, length of hospital stay, and hospital mortality.

### Statistical analysis

All encounters were divided into three subgroups, according to their location of treatment (IMCU only, ICU only, or both [IMCU/ICU]). In cases of normal distribution of the data, the results are expressed as mean ± standard deviation (SD) and, in cases where data were not normally distributed, as median (interquartile range [IQR]). Receiver operating characteristic curves (ROC) were used for calculation of the predictive validity of the SIRS criteria, qSOFA score, and SOFA score. The primary aims of these analyses were defined as the identification of suspected infection and the prediction of mortality, assessed by means of the area under the ROC curve (AUCROC). Furthermore, sensitivity and specificity of both primary aims were calculated. AUCROCs were considered to be poor at 0.51–0.69, adequate at 0.7–0.79, sufficient at 0.8–0.89, and excellent at 0.9 or higher. AUCROCs are displayed with the 95% confidence interval. Data were tested for statistically significant differences using chi-squared test or Fisher’s exact test, when appropriate. A two-tailed value of *p* < 0.05 was considered to be statistically significant. All statistical analyses were performed using the R statistical software version 3.5.1 (www.r-project.org).

## Results

### Characteristics of the study cohorts

**Fig. 1:**
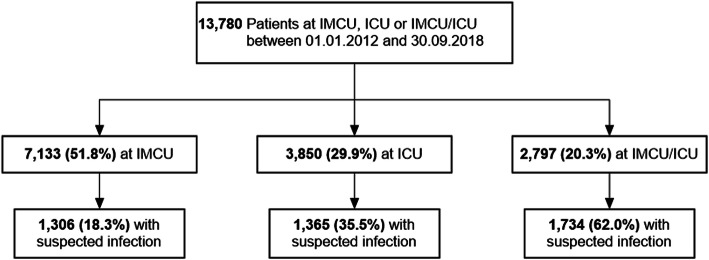
Composition of the different groups. ICU, intensive care unit; IMCU, intermediate care unit

For the observational period, 13,780 patients were identified. Of these, 7133 (51.8%) were treated only at the IMCU, 3850 (27.9%) at the ICU, and 2797 (20.3%) at both the ICU and IMCU (Fig. 1). Patients’ characteristics, underlying departments, and outcome parameters are shown in Table 1. Overall, 458 (3.3%) subjects died within the observation period (IMCU 45 [0.6%]; ICU 250 [6.5%]; IMCU/ICU 163 [5.8%]). Suspected infections were identified in 4405 (32.0%) patient encounters (IMCU 1306 [18.3%]; ICU 1365 [35.5%]; ICU/IMCU 1734 [62.0%]; Fig. 1).

**Table 1:** Basic patient characteristics

Parameter	IMCU	ICU	IMCU/ICU	All
**Age (years)**	61 [41–76]	64 [52–75]	69 [57–78]	64 [49–76]
**BMI (kg/m**^**2**^**)**	26.8 [23.9–31.0]	26.2 [23.4–29.8]	26.7 [23.9–30.9]	26.6 [23.7–30.7]
**APACHE II**	4 [0–12]	12 [0–19]	18 [14–23]	10 [0–17]
**Invasive ventilation**	1.9%	52.3%	67.0%	29.2%
**Need for catecholamines**	4.3%	38.0%	57.1%	24.4%
**Hospital stay**	3.12 ± 11.91	4.80 ± 12.34	23.11 ± 33.79	7.65 ± 20.22
**Hospital mortality**	0.6%	6.5%	5.8%	3.3%
**Infection**	18.3%	35.5%	62.0%	32.0%

Data are expressed as median with interquartile range (IQR), percentage, or, if normally distributed, as mean with standard deviation (±)

*APACHE II* Acute Physiology and Chronic Health Evaluation, *BMI* Body mass index, *ICU* intensive care unit, *IMCU* Intermediate care unit

### Performance of clinical scores in the IMCU

Among 1306 IMCU patients with suspected infection, 1023 (78.3%) fulfilled at least two positive SIRS criteria. Furthermore, a SOFA score increase was detected in 65 (5.0%) cases, while qSOFA scoring was positive in 735 (56.3%) patients.

**Fig. 2:**
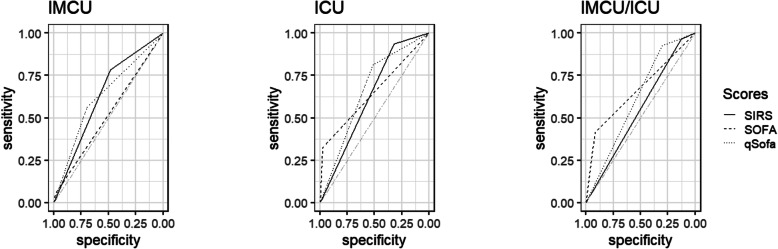
Predictive validity for suspected infection using clinical scores. Receiver operating characteristic curves for positive SIRS criteria, SOFA score, and qSOFA score are pictured. ICU, intensive care unit; IMCU, intermediate care unit; qSOFA, quick Sequential Organ Failure Assessment; SIRS, systemic inflammatory response syndrome; SOFA, Sequential Organ Failure Assessment

Overall, the predictive performance of the scores of interest was low. However, compared to the SOFA score, the SIRS criteria and qSOFA score performed superiorly regarding their discrimination between suspected infection and the use of broad-spectrum antibiotics (SIRS: AUCROC = 0.63 [0.62–0.65]; SOFA: AUCROC = 0.52 [0.51–0.53]; qSOFA: AUCROC = 0.63 [0.62–0.65]; SIRS vs. SOFA: *p* < 0.001; qSOFA vs. SOFA: *p* < 0.001; SIRS vs. qSOFA: *p* = 0.833; Fig. 2). While the highest sensitivity for the detection of presumed sepsis was achieved by means of the SIRS criteria, the maximum specificity was found with the SOFA score (Table 2).

**Table 2:** Sensitivity and specificity of clinical scores for infection

Parameter	IMCU	ICU	IMCU/ICU
**Sensitivity of SIRS**	0.78	0.93	0.97
**Specificity of SIRS**	0.48	0.32	0.13
**Sensitivity of SOFA**	0.05	0.33	0.42
**Specificity of SOFA**	0.99	0.97	0.92
**Sensitivity of qSOFA**	0.56	0.81	0.93
**Specificity of qSOFA**	0.70	0.51	0.30

*ICU* Intensive care unit, *IMCU* Intermediate care unit, *SIRS* Systemic inflammatory response syndrome, *SOFA* Sequential Organ Failure Assessment, *qSOFA* Quick Sequential Organ Failure Assessment

**Fig. 3:**
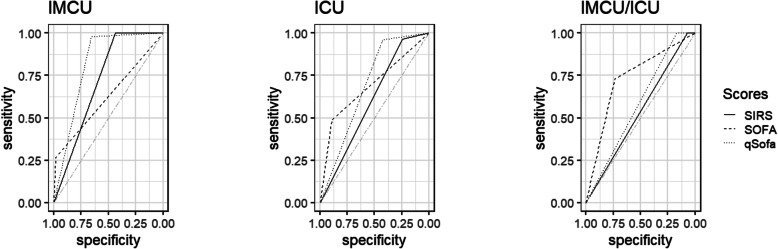
Predictive validity for hospital mortality using clinical scores. Receiver operating characteristic curves for positive SIRS criteria, SOFA score, and qSOFA score are pictured. ICU, intensive care unit; IMCU, intermediate care unit; qSOFA, quick Sequential Organ Failure Assessment; SIRS, systemic inflammatory response syndrome; SOFA, Sequential Organ Failure Assessment

All IMCU patients with suspected infection who died (45 [3.4%]) fulfilled at least two SIRS criteria, while the SOFA score was positive in 12 (26.7%) lethal cases and the qSOFA score in 44 (97.8%) of those who died. The highest predictive validity for hospital mortality was achieved by calculating the qSOFA score, while SIRS criteria and SOFA score performed significantly inferiorly regarding their predictive validity (SIRS: AUCROC = 0.72 [0.71–0.72]; SOFA: AUCROC = 0.63 [0.56–0.69]; qSOFA: AUCROC = 0.82 [0.79–0.84]; SIRS vs. SOFA: *p* = 0.006; qSOFA vs. SOFA: *p* < 0.001; SIRS vs. qSOFA: *p* < 0.001; Fig. 3). SIRS criteria and qSOFA score reached high sensitivity and low specificity regarding mortality, while the SOFA score revealed contrary results (Table 3).

**Table 3:** Sensitivity and specificity of clinical scores for mortality

Parameter	IMCU	ICU	IMCU/ICU
**Sensitivity of SIRS**	1.00	0.96	1.00
**Specificity of SIRS**	0.44	0.24	0.07
**Sensitivity of SOFA**	0.27	0.49	0.73
**Specificity of SOFA**	0.98	0.89	0.74
**Sensitivity of qSOFA**	0.98	0.96	1.00
**Specificity of qSOFA**	0.65	0.42	0.17

*ICU* Intensive care unit, *IMCU* Intermediate care unit, *SIRS* Systemic inflammatory response syndrome, *SOFA* Sequential Organ Failure Assessment, *qSOFA* Quick Sequential Organ Failure Assessment

### Performance of clinical scores in the ICU

Of 1635 ICU encounters with suspected infection, a SOFA score increase was identified in 446 (32.7%) encounters, while qSOFA scoring was positive in 1111 (81.4%) cases. In 1276 (93.5%) encounters, at least two SIRS criteria were fulfilled.

Overall, the discriminative power for the identification of patients receiving broad-spectrum antibiotic treatment in the cohort of patients with suspected infection was poor (SIRS: AUCROC = 0.63 [0.62–0.64]; SOFA: AUCROC = 0.65 [0.64–0.66]; qSOFA: AUCROC = 0.66 [0.65–0.68]; SIRS vs. SOFA: *p* = 0.008; qSOFA vs. SOFA: *p* = 0.098; SIRS vs. qSOFA: *p* < 0.001; Fig. 2). SIRS criteria and qSOFA score were highly sensitive but not specific for presumed sepsis, while contrary results were demonstrated for the SOFA score (Table 2).

Overall, 250 (15.3%) ICU patients with suspected infection deceased. A majority of these subjects showed positive SIRS criteria (241 [96.4%]) and qSOFA score (240 [96.0%]), while positive SOFA was detected only in 122 (48.8%) encounters. SIRS criteria and qSOFA score reached high sensitivity but low specificity regarding the prediction of mortality, while SOFA score revealed contrary results (Table 3). Overall, the predictive validity of all included scores was poor. However, compared to SIRS criteria, SOFA and qSOFA scores performed superiorly regarding the prediction of mortality (SIRS: AUCROC = 0.60 [0.59–0.62]; SOFA: AUCROC = 0.69 [0.66–0.72]; qSOFA: AUCROC = 0.69 [0.68–0.71]; SIRS vs. SOFA: *p* < 0.001; qSOFA vs. SOFA: *p* = 0.92; SIRS vs. qSOFA: *p* < 0.001; Fig. 3).

### Clinical scores in patients treated at the ICU and IMCU

Among the 1734 (62.0%) encounters with suspected infection in patients who were admitted to the IMCU and ICU, 1676 (96.7%) showed at least two positive SIRS criteria, while SOFA and qSOFA scores were positive in 721 (41.6%) and 1607 (92.7%) encounters, respectively.

The predictive validity for presumed sepsis of all scores was poor (SIRS: AUCROC = 0.55 [0.54–0.56]; SOFA: AUCROC = 0.67 [0.65–0.68]; qSOFA: AUCROC = 0.61 [0.60–0.63]; SIRS vs. SOFA: *p* < 0.001; qSOFA vs. SOFA: *p* < 0.001; SIRS vs. qSOFA: *p* < 0.001; Fig. 2). While the SIRS criteria and qSOFA score revealed high grades of sensitivity and low specificity, contrary results were demonstrated for the SOFA score (Table 2).

Moreover, mortality among the encounters with suspected infection on the IMCU/ICU amounted to 163 (9.4%). All of them offered a positive qSOFA score and at least two positive SIRS criteria (163 [100%]), while the SOFA score was increased in 119 (73.0%) encounters.

Regarding hospital mortality, the SIRS criteria and qSOFA score revealed only poor predictive validity, whereas the SOFA score was predictive for the patients’ death (SIRS: AUCROC = 0.54 [0.53–0.54]; SOFA: AUCROC = 0.73 [CI, 0.70–0.77]; qSOFA: AUCROC = 0.59 [0.58–0.59]; SIRS vs. SOFA: *p* < 0.001; qSOFA vs. SOFA: *p* < 0.001; SIRS vs. qSOFA: *p* < 0.001; Fig. 3). SIRS criteria and qSOFA score reached high sensitivity but low specificity regarding mortality, whereas the SOFA score performed adequately (Table 3).

## Discussion

The rapid identification of sepsis serves as the basis for its successful management. According to the current recommendations of the Surviving Sepsis Campaign, the SOFA score should be used as a predictive tool for the detection of sepsis as well as for the risk stratification of critically ill patients. In addition, the qSOFA score has been introduced for the identification of septic patients outside of the ICU [7, 20, 21]. However, to our knowledge, both scores have not been evaluated in the context of surgical IMCU patients. Therefore, this is the first study comparing the predictive value for presumed sepsis of the SOFA and qSOFA scores, as well as the 1992 defined SIRS criteria, in a large cohort of 13,780 surgical IMCU and ICU patients of a tertiary university hospital.

Overall, among encounters with suspected infection in IMCU patients, none of the analyzed scoring tools showed sufficient predictive validity for severe infection (defined as the use of broad-spectrum antibiotics), whereas the qSOFA score was able to predict mortality in a sufficient manner. Interestingly, even though the assessment with the historical SIRS criteria does not meet the current practice guidelines, they were more predictive than the SOFA score within the IMCU patient cohort. Furthermore, among ICU patients as well as patients who underwent a combined IMCU and ICU treatment, all analyzed scoring systems failed to provide sufficient validity for the prediction of infection and mortality. Only in patients who underwent a combined IMCU and ICU treatment the SOFA score was able to adequately predict mortality.

At first glance, these study results might be surprising. However, in comparison to previous findings, the performance of the qSOFA score and the SIRS criteria remain agreeable. The qSOFA score was first developed and validated by Seymour et al., who analyzed 148,907 unselected patient encounters with suspected infection, consisting of a validation cohort of 7932 ICU and 66,522 non-ICU patients. With the exception of the SOFA score, the predictive value of the qSOFA and SIRS criteria could be matched to our study results within the ICU cohort (AUCROC SOFA 0.74 vs. 0.52; AUCROC qSOFA 0.66 vs. 0.63; AUCROC SIRS 0.64 vs. 0.63) [7]. Their findings have been validated in several studies featuring large numbers of patients (Table 4), resulting in a varying performance of the mentioned scores. However, it has to be stressed that originally Seymour et al. aimed to validate the qSOFA and SOFA scores as predictors for mortality and not for the identification of sepsis. The Sepsis-3 definition indicates that, due to their predictive value for mortality, both scores can be used for sepsis risk stratification (either at the ICU or non-ICU), but also emphasizes that the underlying data was derived from retrospective studies and requires further validation. However, until now, no prospective data, with sufficient numbers of patients, is available.

**Table 4:** Overview studies regarding clinical criteria

Author	Patients	Collective	Primary outcome	Suspected infection	SIRS	SOFA	qSOFA
**Lo et al.** [20]	*n* = 380,920	Mixed	Mortality	No	n.a.	n.a.	0.68
**Kovach et al.** [21]	*n* = 10,981	ED; ICU; mixed	Mortality	Yes	0.79	0.90	0.84
**Seymour et al.** [7]	*n* = 7932	ICU; mixed	Mortality	Yes	0.64	0.74	0.66
**Zhang et al.** [23]	*n* = 5109	Surgical ICU	Mortality	No	0.95	0.96	0.95
**Falcao et al.** [31]	*n* = 3008	Surgical ICU	Mortality	No	n.a.	0.742	n.a.
**Gando et al.** [25]	*n* = 1045	ED; mixed	Infection	Yes	0.647	n.a.	0.582
**Basile-Filho et al.** [32]	*n* = 847	Surgical ICU	Mortality	No	n.a.	0.791	n.a.
**Mungan et al.** [33]	*n* = 233	Surgical ICU	Mortality	No	n.a.	0.631	n.a.
**Innocenti et al.** [34]	*n* = 135	ED-HDU; mixed	Mortality	Yes	n.a.	0.80	n.a.

*ICU* Intensive care unit, *ED* Emergency department, *HDU* High-dependency unit, *qSOFA* Quick Sequential Organ Failure Assessment, *SIRS* Systemic inflammatory response syndrome, *SOFA* Sequential organ failure assessment

Lo et al. performed a literature review and retrospective data analysis of 380,920 patients, demonstrating an AUCROC of 0.68 for the predictive value of in-hospital mortality for the qSOFA score, which is in line with our study findings in surgical ICU and IMCU patients [20]. Furthermore, a meta-analysis of 229,480 patients compared the qSOFA score and SIRS criteria for their ability to predict patient mortality and revealed only a slightly better performance of the qSOFA score, which supports the findings of our study [22]. However, some studies revealed a high power for the prediction of mortality: Kovach et al. analyzed hospital mortality in a retrospective data set of 3749 surgical and medical ICU patients with suspected infection, while Zhang et al. investigated retrospectively 5109 cardiac surgical patients, with both studies resulting in AUCROC > 0.8 for the prediction of mortality by using the SOFA and qSOFA scores [21, 23]. However, it must be highlighted that, contrary to our approach, the patients of Kovach’s study were adjusted for a baseline risk factor for death, which increased the predictive quality of the SOFA score, while Zhang et al. only included cardiac surgical patients, which are hardly comparable with the sources of systemic inflammation in our study. During cardiac surgery, systemic inflammation is mainly induced by cardiopulmonary bypass, which leads to strong activation of the inflammatory response through the blood’s foreign surface contact with the components of the heart-lung machine, reperfusion injury/reperfusion injury [24]. Contrarily, local surgical trauma is causative for the onset of inflammation during non-cardiac surgery.

Even though qSOFA and SOFA scores are widely accepted as tools for the identification of septic patients, they failed to predict suspected infection in each individual group of patients in our study. These findings are supported by Krebs et al., who also evaluated the qSOFA and SOFA scores as well as the SIRS criteria in 1942 prospective patient days within a cohort of surgical trauma ICU patients, concluding that all scores failed to predict the development of new infections [17]. But, also in an out-of-ICU setting, a failure of the qSOFA score (and SIRS criteria) has already been described in a collective of patients attending the emergency room (*n* = 1045) [25]. Moreover, another large retrospective analysis failed to prove a high predictive power of the qSOFA score and the SIRS criteria in patients admitted to the emergency department [26].

These opposing results might be partially explainable, as already discussed above, by the choice of the study population, which might strongly influence the study results because only postsurgical patients were investigated in our study, in contrast to medical and surgical patients in the underlying study. Further, the variable predictive validity between the studies might be caused by the differences in the study designs. Considering the original publication of Seymour et al., the lower predictive capacity of the SOFA score in our study might be caused by the varying definition of suspected infection. While it was defined as the combination of antibiotics and body fluid cultures by Seymour et al., the administration of broad-spectrum antibiotics was used in our approach. It has to be noted that the prescribing behavior of antibiotics varies between physicians depending on their clinical experience, qualification, and specialty. Charani et al. compared the antibiotic prescribing between medical and surgical specialties. Besides more frequent and longer prescription, antibiotics were more likely to be escalated in surgical patients [27]. A recent systemic review offers a potential explanation for these findings by identifying nine determinants that influenced antibiotic prescription behavior including the fear of risking an adverse outcome [28]. Surgical patients are challenging when it comes to identifying infectious complications, and the consequences of sepsis are more devastating in these patients which potentially leads to a more liberal application of broad-spectrum antibiotics [29, 30]. This might offer an explanation for the low specificity of the analyzed scores for detecting a presumed sepsis. Furthermore, even in an isolated analysis of studies that only investigated surgical patients, the predictive performance varies strongly: Falcao et al. analyzed 3008 surgical ICU patients and showed a sufficient predictive validity of the SOFA score regarding mortality (AUCROC of 0.74) [31]. Similar results are published by Basile-Filho et al., who revealed an AUCROC of 0.79 by using the SOFA score for the prediction of mortality within 847 surgical ICU patients [32]. Contrarily, Mungan et al. showed a lower predictive validity of 0.63 of the SOFA score in surgical ICU patients [33]. Although authors of these studies described their population as “surgical patients,” it must be highlighted that their calculations comprised all kinds of surgical patients, independently of their risk for infection, including those without suspicion of infection. By contrast, our study only focused on the investigation of postsurgical patients with suspected infection. In our opinion, this issue is of high relevance because the majority of postsurgical patients following major surgery regularly show clinical signs of systemic inflammation such as tachycardia, fever, and tachypnea, which commonly represent signs of a surgery-induced systemic inflammation rather than an infection. For this reason, it is not only challenging to discriminate between postsurgical sterile systemic inflammation and infection, but the predictive ICU sores might also become distorted into false positive results. This may explain the high sensitivity but low specificity of the qSOFA score and SIRS criteria because both systems include only clinical criteria for easy assessment. Since these criteria are often fulfilled during the postsurgical phase, the chance that they are truly positive is high (sensitivity). On the other hand, this leads to a low rate of true false cases (specificity). Since the SOFA score consists of much more detailed intensive care variables than the qSOFA score and the SIRS criteria, the specificity is higher, but sensitivity remains low. These arguments are in accordance with the findings of Gando et al. as well as Krebs et al., who demonstrated that the SIRS criteria, SOFA score, and qSOFA score were not able to predict sepsis in the emergency department or surgical ICU [17, 25].

These limitations of the ICU scores are of high interest for their use on surgical IMCUs because of the increasing demand of IMCU capacity. Therefore, the importance of the surgical IMCU, as a bridge to the normal ward, is rising. Patients attending the IMCU commonly represent those surgical patients at moderate to high risk of developing postsurgical complications. Analogous to ICU patients, the rapid identification of infectious complications is altered by surgery-induced signs of systemic inflammation, underlining the need for specific IMCU scores. Lacking studies that focus on surgical IMCUs, other high-dependency units (HDUs) (but not ICUs) have to be analyzed for the interpretation of our study results. Innocenti et al. analyzed 3311 patients admitted to HDUs and demonstrated that the SOFA score, in opposition to our results, showed a good discriminatory ability for HDU mortality [34]. However, contrary to our approach, no cutoff values for SOFA scores were used, and no postsurgical patients were included. In our study, the prediction of mortality was sufficient using the qSOFA score in IMCU patients. Another study showed that these scores are also not able to predict infection in the emergency room [25]. Based on the findings of our study, the use of the qSOFA score as a predictor of mortality can be supported, while its predictive power for the detection of suspected infection can be doubted in postsurgical IMCU patients, which might be caused by surgery-induced systemic inflammation.

However, due to the retrospective study design, further prospective studies that include high numbers of postsurgical IMCU patients are necessary to validate these findings. Due to the fact that most critically ill patients are regularly transferred to the IMCU during their medical treatment, a subgroup of these patients was created. The increased APACHE II score reflects the serious illness of the included patients. Since clinical scores were not able to distinguish for suspected infection in this subgroup, severity of disease seems not to improve the quality of these scores. While adequate prediction for mortality was calculated with the SOFA score, this could indicate its better quality in critically ill patients. Since these patients are missing in the ICU subgroup, this could also explain our lower results for the SOFA score in the ICU.

Nevertheless, our study features some limitations. First, this retrospective analysis is not able to draw conclusions regarding the underlying causalities. Second, due to the retrospective design, no sample size calculation was performed. Third, lacking of more specific alternatives, the administration of broad-spectrum antibiotic agents was used as a surrogate for the diagnosis of suspected infection. While the clinical symptoms and inflammatory parameters are physiologically altered by the surgery, body fluid cultures result in negative samples in a majority of cases (e.g., due to the perioperative antibiotic treatment) [28, 35, 36]. Furthermore, even if sepsis was assessed by intensivists, its diagnosis remains subjective [37]. Nonetheless, it has to be highlighted that the administration of a broad-spectrum antibiotic agent serves only as a surrogate for the true presence of sepsis. Fourth, the RAAS was used as a surrogate parameter for GCS < 15 in the absence of the GCS, which is problematical since the qSOFA score was developed and validated with the use of GCS.

## Conclusions

In summary, neither SOFA nor qSOFA score was able to distinguish for suspected sepsis (defined by the application of broad-spectrum antibiotics) in surgical patients, independently of IMCU, ICU, or IMCU/ICU stay. Nevertheless, the qSOFA score revealed sufficient prediction for mortality in the IMCU. Further, as the SOFA score showed the best results regarding mortality in IMCU/ICU patients, its predictive quality depended on the severity of the disease. Summarizing, it remains unclear whether qSOFA or SOFA score should be used in surgical IMCU patients for risk stratification.

## Data Availability

All data generated or analyzed during this study are included in this published article (and its supplementary information files).

## Abbreviations

APACHE II: Acute physiology and chronic health evaluation II
AUROC: Area under the receiver operating curve
GCS: Glasgow coma scale
HDU: High-dependency unit
ICU: Intensive care unit
IQR: Interquartile range
IMCU: Intermediate care unit
qSOFA: Quick sequential organ failure assessment
RAAS: Richmond agitation sedation scale
RR: Respiratory rate
SBP: Systolic blood pressure
SD: Standard deviation
SIRS: Systemic inflammatory response syndrome
SOFA: Sequential organ failure assessment
STROBE: Strengthening the reporting of observational studies in epidemiology
